# The impact of timing and injury mode on induced neurogenesis in the adult mammalian retina

**DOI:** 10.1016/j.stemcr.2023.12.010

**Published:** 2024-01-25

**Authors:** Marina Pavlou, Marlene Probst, Nicolai Blasdel, Aric R. Prieve, Thomas A. Reh

**Affiliations:** 1 Department of Biological Structure, University of Washington, Seattle, WA, USA; 2 Institute for Stem Cells and Regenerative Medicine, University of Washington, Seattle, WA, USA

**Keywords:** neurogenesis, Ascl1, Ascl1-Atoh1, Müller glia, retina, inflammation, injury mode, NMDA, light-damage

## Abstract

Regeneration of neurons has important implications for human health, and the retina provides an accessible system to study the potential of replacing neurons following injury. In previous work, we generated transgenic mice in which neurogenic transcription factors were expressed in Müller glia (MG) and showed that they stimulated neurogenesis following inner retinal damage. It was unknown, however, whether the timing or mode of injury mattered in this process. Here, we explored these parameters on induced neurogenesis from MG and show that MG expressing Ascl1 will generate new bipolar neurons with similar efficiency irrespective of injury mode or timing. However, MG that express Ascl1-Atoh1 produce a new type of retinal ganglion-like cell after outer retinal damage, which is absent with inner retinal damage. Our data suggest that although cell fate is primarily dictated by neurogenic transcription factors, the inflammatory state of MG relative to injury can influence the outcome of induced neurogenesis.

## Highlights

• Light damage stimulates neurogenesis from MG expressing proneural factors
• The timing of injury does not affect MG reprogramming with Ascl1 or Ascl1-Atoh1
• MG expressing Ascl1 will give rise to bipolar neurons irrespective of injury mode
• Injury mode affects the types of neurons obtained from MG expressing Ascl1-Atoh1

## Graphical abstract

**Figure:**
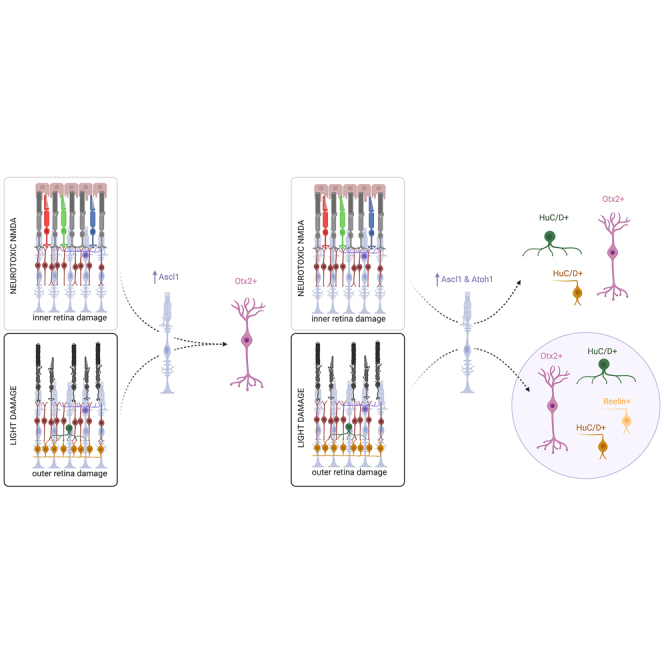

## Teaser

In this article, Reh and colleagues show that inducing the expression of proneural transcription factors stimulates Müller glia to generate new neurons after either inner or outer retinal damage. Although the fate of glial-derived neurons is primarily dictated by the transcription factors, it is influenced by the type of retinal injury and degree of glial inflammation.

## Introduction

The ability to regenerate tissues after an injury or aging has attracted widespread interest from both basic and medical research. Although some of our tissues are able to regenerate, such as the liver (Taub, 2004), damage to the central nervous system (CNS) in mammals leads to permanent neuronal death and functional loss. Some vertebrates, however, retain their ability to regenerate neurons (Sharma and Ramachandran, 2022; Todd and Reh, 2022). Zebrafish, for example, are able to regenerate many areas of the CNS, including the neural retina (Todd and Reh, 2022; Wan and Goldman, 2016), leading to the functional restoration of vision (Hammer et al., 2022). In the zebrafish retina, Müller glia (MG) respond to injury by giving rise to multipotent progenitor cells that reactivate developmental pathways. These progenitors proliferate and give rise to new neurons that repopulate the injured tissue (Wan and Goldman, 2016). This mechanism is lost in the mammalian retina, and several groups have been developing approaches to reinstate this regenerative ability in adult mammalian MG.

We can mimic some aspects of the naturally occurring regeneration in mice by expressing proneural transcription factors (TFs) in adult MG. We have shown that the expression of Ascl1, either alone or in combination with other TFs in adult mammalian MG, followed by an acute retinal injury, causes the MG to acquire a progenitor-like state and generate functional neurons (Jorstad et al., 2017; Todd et al., 2021; 2022). The types of neurons generated by the MG vary depending on the specific TFs expressed in the cells; Ascl1 alone primarily induces bipolar cell production, whereas the combinations Ascl1-Atoh1 or Isl1-Pou4f2-Ascl1 (IPA) reprogram MG mostly into retinal ganglion cell (RGC)–like neurons. In the majority of our reprogramming paradigms, neurogenesis from MG requires not only the expression of pioneering factors such as Ascl1 but also retinal injury.

Similarly, in fish, the process of regeneration is also stimulated by retinal injury and requires Ascl1 expression in MG (Fausett et al., 2008). There have been a number of mechanical, chemical, and light-induced injuries used to ablate the entire retina, or specific neuronal classes, to study how the retina responds to each lesion (see Table 1 of Sharma and Ramachandran, 2022). In zebrafish, MG respond to both N-methyl-d-aspartate (NMDA) and light-damage by upregulating cell cycle and protein biosynthesis genes as they transition to multipotent progenitors (Hoang et al., 2020). These progenitors will generate all of the classes of retinal neurons, regardless of which cells died during the injury (Powell et al., 2016), although some bias to regenerate the cell types lost has been reported in the case of red and UV cone photoreceptors (D’Orazi et al., 2020). This may be the case in fish; however, it is not known whether induced neurogenesis in mice is affected by the type of retinal injury. In our previous work, we only tested neurotoxic NMDA as a mode of injury and saw that damage was necessary to stimulate neurogenesis from MG that express Ascl1 alone or IPA (Jorstad et al., 2017; Todd et al., 2022). Therefore, we asked whether a different mode of injury would be sufficient to stimulate neurogenesis from MG in adult mice.

In addition to the type of injury, the timing of injury relative to the expression of proneural factors may play an important role in the regenerative response of mammalian MG. The transient inflammatory state induced in the MG after injury in zebrafish and mice may be necessary to initiate the neurogenic process (Iribarne and Hyde, 2022) and may predispose the fate of neurons derived from inflamed MG. In fish, the regeneration pathways are naturally turned on within 24 h of injury (Hoang et al., 2020), and it is not known whether altering the timing of this response would change the outcome. In mice, in which neurogenesis is artificially stimulated with proneural TFs such as Ascl1, we induce retinal injury with a toxic dose of intravitreal NMDA after the expression of the TF in MG; however, it remains unclear whether mammalian MG can become neurogenic if Ascl1 is induced following injury. Deciphering the importance of timing could greatly affect the potential translation of glia-to-neuron reprogramming in human patients, because a regenerative therapy would need to stimulate neurogenesis after retinal damage has already occurred.

To test the effects of a different injury mode, we used light-damage to ablate photoreceptors and asked whether this would provide a sufficient stimulus to drive neurogenesis from MG expressing Ascl1 or Ascl1-Atoh1. We also asked whether the timing of TF expression either before or after light-damage would change the neurogenic potential of MG in the adult mouse retina. The results show (1) that both light-damage and neurotoxic NMDA provide a sufficient stimulus to drive the process of neurogenesis in MG that express Ascl1; (2) that the expression of Ascl1 after retinal injury induces neurogenesis in the MG; and (3) the types of neurons generated by the MG depend primarily on the TF combination used for reprogramming but can be influenced by the mode of injury. Our results overall support a model in which the potent effects of TF expression can be modulated by factors in the damaged retinal microenvironment.

## Results

### Injury mode does not change the outcome of MG reprogramming with Ascl1

We used the transgenic mice from our previous study (Jorstad et al., 2017), which express Ascl1 and green fluorescent protein (GFP) in MG in a tamoxifen-inducible manner, to assess the effects of injury mode on induced neurogenesis. In the past, we would intravitreally deliver a neurotoxic dose of NMDA, which would cause inner retinal damage. To induce outer retinal damage in our present study, we subjected the mice to a light-damage paradigm, in which animals were exposed to bright white light for 6 h. This treatment caused severe photoreceptor degeneration, primarily in the central retina, within 1 week post light-damage (Figure S1A).

**Figure 1:**
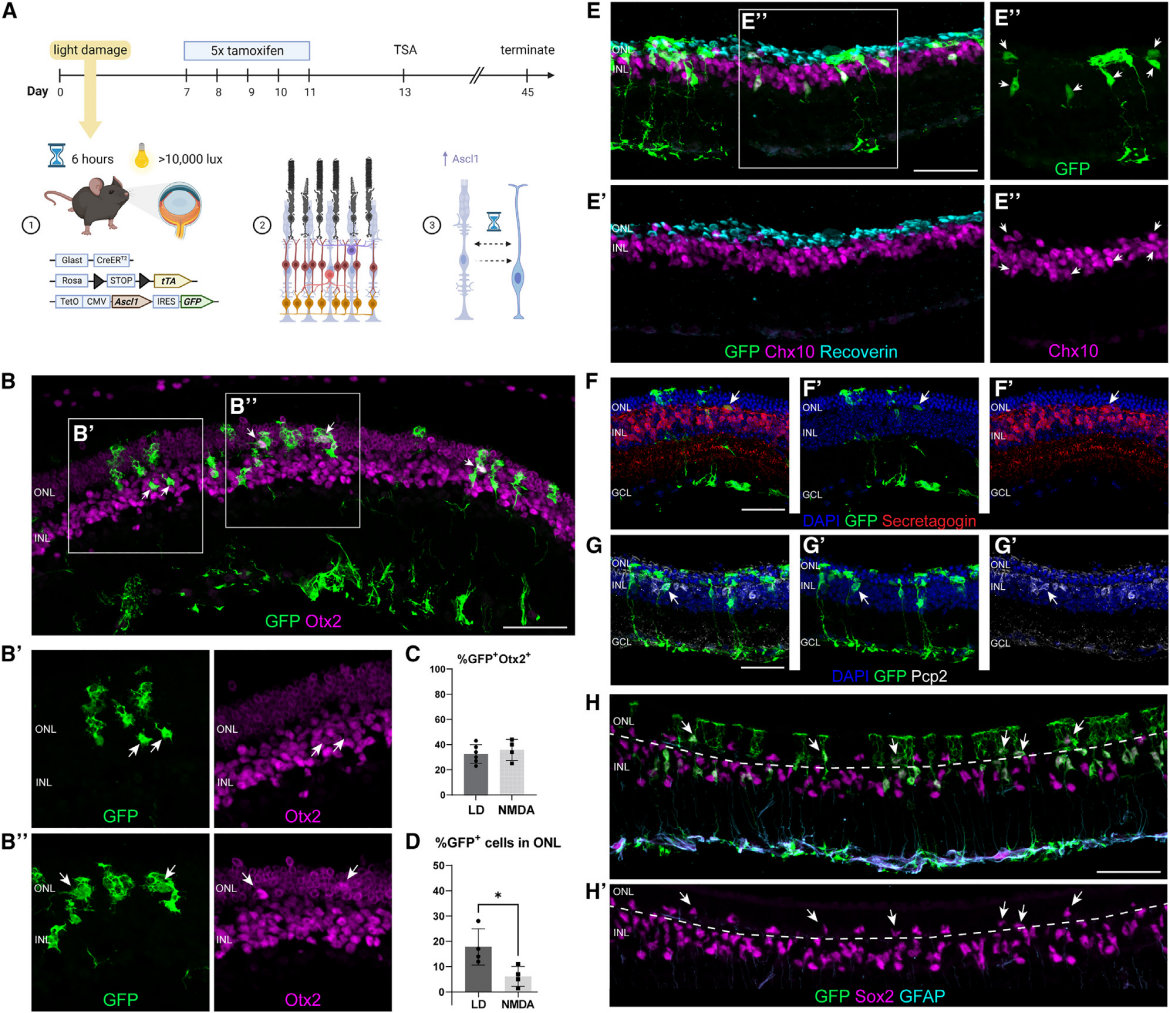
The impact of light damage on reprogramming MG with Ascl1 (A) Schematic overview of the experimental time line in which transgenic mice expressing Ascl1 specifically in MG in a tamoxifen-inducible manner undergo light-damage. (B–B″) Fluorescent images of retinal cross-section showing lineage-traced GFP^+^Otx2^+^ cells in the (B′) INL and (B″) ONL. (C) Quantification of double-labeled GFP^+^Otx2^+^ cells after light-damage and NMDA injuries. (D) Quantification of GFP^+^ cells in the ONL after light-damage and NMDA injuries. (E–E″) Fluorescent images of retinal cross-section showing lineage-traced cells expressing GFP colabeled with Chx10 (white arrows) but not recoverin. (F–F′) Fluorescent images of retinal cross-section showing lineage-traced GFP^+^Secretagogin^+^ cell (white arrow). (G–G′) Fluorescent images of retinal cross-section showing lineage traced GFP^+^Pcp2^+^ cell (white arrow). (H–H′) Fluorescent images of retinal cross-section showing lineage-traced GFP^+^Sox2^+^ cells (white arrows) but not glial fibrillary acidic protein (GFAP) in the ONL; scale bar: 50 μm. Bar graphs (n ≥ 3 animals) with SEM error bars and unpaired t test analysis where ^∗^p < 0.05.

To determine whether light-damage provides sufficient stimulus for neurogenesis from MG, we induced Ascl1 expression in MG using tamoxifen 1 week post light-damage, followed by intravitreal administration of trichostatin-A (TSA) to favor chromatin accessibility as in our previous work (Figure 1A). We analyzed the retinas for evidence of neurogenesis 3 weeks post-TSA and found glia-derived neurons. Lineage-traced GFP^+^ cells with neuron morphology were found in both the inner nuclear layer (INL) (Figures 1B–1B′, arrows) and degenerated outer nuclear layer (ONL) (Figure 1B″, arrows); many of these cells expressed Otx2, which is present in photoreceptors and bipolar cells in the retina. Many of the Otx2^+^ MG-derived cells expressed the bipolar cell markers Chx10/Vsx2 (Figures1E–1E″), and even mature bipolar markers such as Secretagogin and Pcp2 (Figures 1F and 1G, arrows); we did not find any cells that were GFP^+^ and expressed the photoreceptor marker Recoverin (Figure 1E′).

To compare the efficiency of induced neurogenesis after light-damage versus NMDA injury, we quantified the percentage of glia-derived Otx2^+^ neurons in both types of injury; we found that approximately 30%–40% of the MG-derived cells express the bipolar marker Otx2 (Figure 1C). The data show that inducing neurogenesis with Ascl1 produces primarily bipolar cells after either outer or inner retinal damage, suggesting that the mode of injury did not affect the fate of new neurons obtained from Ascl1-expressing MG. However, there was a significant increase in the number of lineage-traced GFP^+^ cells that migrated to the ONL following light-damage compared to NMDA (Figure 1D). This result suggests that photoreceptor death provides a different stimulus for cell migration than RGC death. In addition to Otx2^+^ MG-derived neurons, Sox2^+^ MG/progenitors are also more likely to translocate to the ONL in the light-damaged retinas (Figures 1H–1H′, arrows). Not all MG nuclei in the ONL are lineage traced with GFP, suggesting that this response is not exclusive to Ascl1-expressing MG (Figure 1H). Furthermore, although GFP^+^ cells were also found in the outer plexiform layer margins, they were not Calbindin^+^ horizontal cells (Figure S1B).

### MG reprogramming with Ascl1 is not affected by timing of Ascl1 expression relative to injury

In our initial experiment, we induced Ascl1 expression in the MG following light-damage, whereas in our prior work, we would first drive Ascl1 expression in the MG before using NMDA to damage the inner retina. Therefore, to better understand whether the timing of Ascl1 expression relative to injury would have an effect on neurogenesis, we performed another experiment in which Ascl1 was expressed in MG before light-damage (Figure S2A). The results were similar to what was described above i.e., neurogenesis from MG was comparable regardless of whether Ascl1 was induced in the MG before or after light-damage (Figure S2B). Glia-derived GFP^+^Otx2^+^ neurons were found in areas of both severe ONL degeneration (Figures S2C–S2C′) and areas where ONL thickness was maintained (Figures S2D–S2D′). Thus, the timing of injury relative to the induction of Ascl1 in the MG, at least within the parameters we tested, does not affect the outcome of the reprogramming process. This suggests that the injury stimulus required for Ascl1-expressing MG to give rise to neurons is maintained for at least 1 week following outer retinal damage.

**Figure 2:**
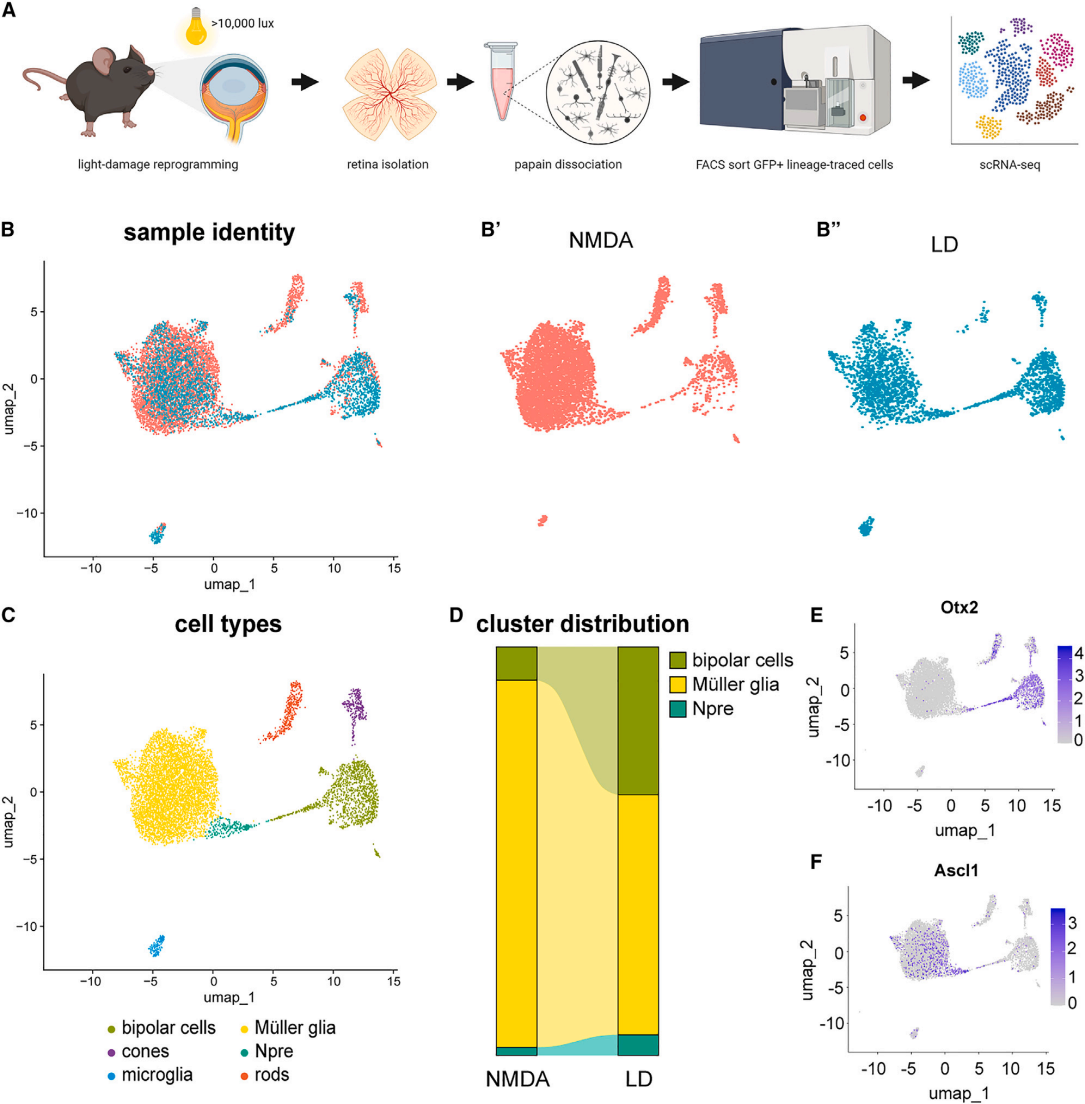
The impact of light damage on the transcriptome of reprogrammed MG with Ascl1 (A) Schematic overview of the experimental time line in which light-damaged retinas were isolated and lineage-traced GFP^+^ cells were sorted for scRNA-seq. (B–B″) UMAP of integrated Seurat objects from sequencing runs of sorted cells from light-damaged and NMDA-treated retinas showing the representation of the (B′) NMDA sample and (B″) light-damaged sample. (C) UMAP of integrated Seurat objects split into clusters of cell types based on transcript signatures. (D) Alluvium plot of subset cell types originating from the NMDA and light-damaged dataset. (E) Feature plot of Otx2 transcripts in the UMAP of integrated Seurat objects. (F) Feature plot of Ascl1 transcripts in the UMAP of integrated Seurat objects.

To better characterize whether the mode of injury affects the types of neurons generated by the MG-derived progenitors and the efficiency of neurogenesis, we performed single-cell RNA sequencing (scRNA-seq) of GFP^+^ sorted cells after light-damage (Figure 2A). We integrated the two Seurat objects from sequencing runs on NMDA-treated cells and light-damaged cells into a single uniform manifold approximation and projection (UMAP) (Figure 2B). The integrated UMAP, in which each dot is a single cell, shows each cluster containing cells from both conditions (Figures 2B′–2B″). The clusters were defined based on their transcript composition, namely MG, neurogenic progenitors (Npre), bipolar cells, rods, cones, and microglia (Figure 2C). From this we can corroborate our histology results, since we find both MG-derived progenitors and bipolar cells in the light-damaged sample. Thus, light-damage provides a sufficient stimulus to trigger neurogenesis in Ascl1-expressing MG, even when preceding the induction of Ascl1.

In addition to MG and MG-derived Otx2^+^ neurons, we also detected clusters of cells that express rod and cone photoreceptor genes. In previously published studies of MG reprogramming, we have reported small numbers of rods and cones (Todd et al., 2021; 2022). Although these cells may be derived from MG, like the bipolar cells, it is also possible that these cells were not MG derived, but rather carry over from our sorting pipeline, because we have yet to detect 5-ethynyl-2′-deoxyuridine^+^ (EdU^+^) lineage-traced photoreceptors in histology. We have however detected lineage-traced GFP+EdU+Otx2+ cells (Figure S4A), confirming the genesis of new bipolar cells. Consistent with the possibility of carry over is the fact there are fewer rod photoreceptors in the light-damaged dataset (Figure 2B’’).

By integrating the light-damaged dataset with our datasets from previous publications, we could directly compare the efficiency of reprogramming in these two different injury paradigms and the types of neurons generated by the Ascl1-expressing MG. Interestingly, when comparing the transcriptome of cells obtained from either injury mode, we find that neurogenesis (Npre and bipolar cells) occurred more efficiently in light-damaged retinas since there were more lineage-traced cells that remained MG after NMDA than light-damage (Figure 2D). When plotting the Ascl1 and Otx2 transcripts on the integrated UMAP, we see a clear transition of the Otx2^+^ cluster emerging from the Ascl1^+^ cluster (Figures 2E and 2F), indicating a similar trajectory of reprogramming for both injury modes.

### Injury mode shifts the outcome of MG reprogramming with Ascl1-Atoh1

The results described above indicate that light-damage can provide a sufficient stimulus for neurogenesis from Ascl1-expressing MG. In previous studies, we have found that expressing alternative TF combinations in MG can lead to the genesis of other retinal subtypes. For example, expressing both Ascl1 and Atoh1 in MG can significantly increase the rate of neurogenesis and shift the type of neurons from Otx2^+^ bipolar cells to Hu protein C and D (HuC/D^+^) RGC-like cells and amacrine cells (Todd et al., 2021).

**Figure 3:**
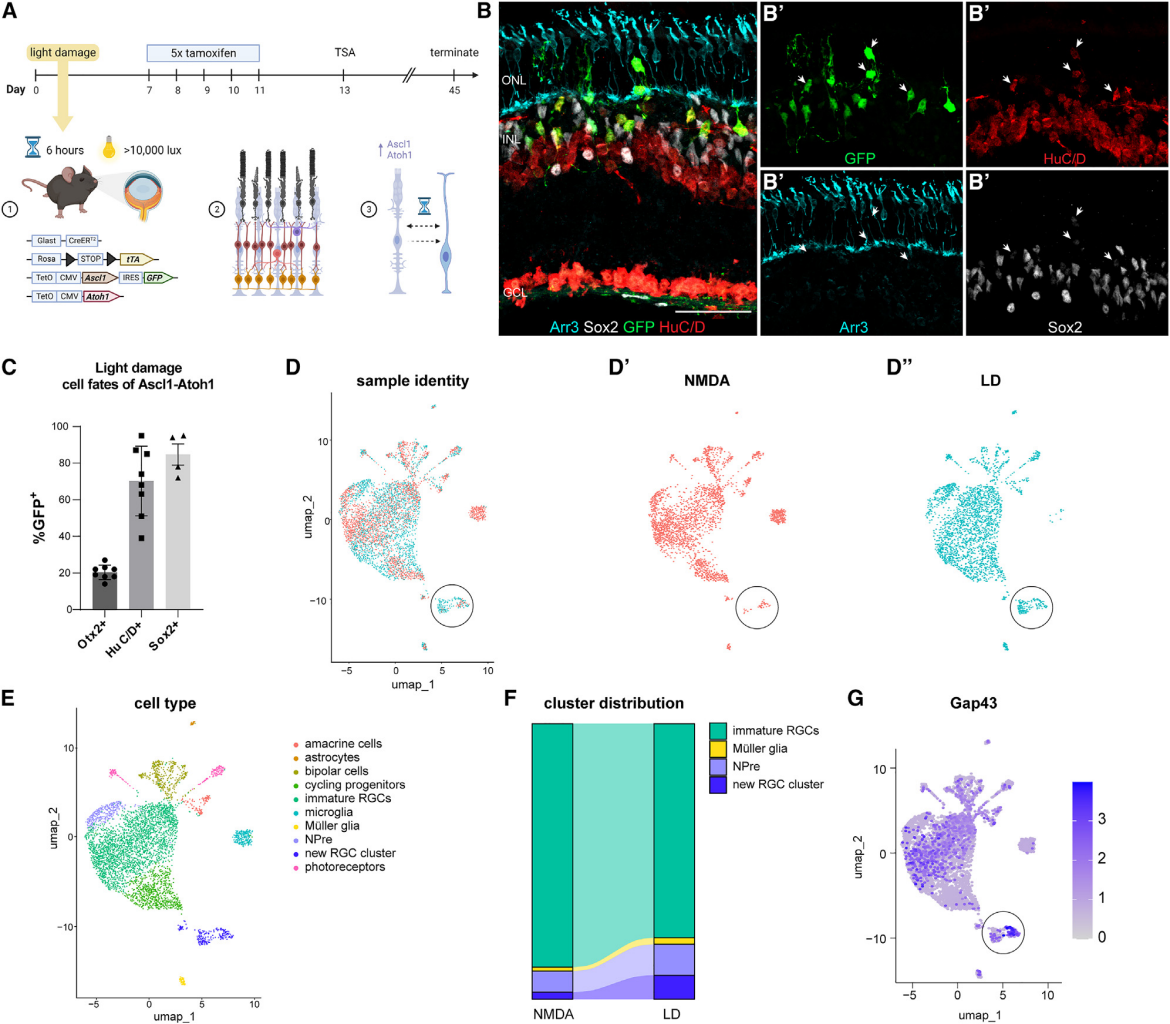
The impact of light damage on reprogramming MG with Ascl1-Atoh1 (A) Schematic overview of the experimental time line in which transgenic mice expressing Ascl1-Atoh1 specifically in MG in a tamoxifen-inducible manner undergo light-damage. (B–B′) Fluorescent images of retinal cross-section showing lineage-traced cells with GFP colabeled with HuC/D and Sox2 (white arrows) but not Arr3. (C) Quantification of lineage-traced glia colabeled with neuronal markers Otx2 and HuC/D and glial marker Sox2 after light-damage. (D–D″) UMAP of integrated Seurat objects from sequencing runs of sorted cells from light-damaged and NMDA-treated retinas showing the representation of the (D′) NMDA sample and the (D″) light-damaged sample. (E) UMAP of integrated Seurat objects split into clusters of cell types based on transcript signatures. (F) Alluvium plot of subset cell types originating from the NMDA and light-damaged dataset. (G) Feature plot of Gap43 transcripts in the UMAP of integrated Seurat objects. Scale bar: 50 μm; bar graph (n ≥ 4 animals) with SEM error bars.

To test whether the mode of injury affects the types of neurons generated with the Ascl1-Atoh1 combination, we exposed transgenic mice that express Ascl1-Atoh1 in MG to the same light-damage paradigm as above. One week later, we induced Ascl1-Atoh1 expression in MG using tamoxifen, followed by an intravitreal injection of TSA; we analyzed the retinas 3 weeks later (Figure 3A) for evidence of MG-derived neurons. The retinas of treated animals showed overall ONL thinning, with cones and inner retinal layers largely intact (Figure S3A). Lineage-traced GFP^+^ cells were found across all of the retinal layers (Figure 3B), and as with the NMDA injury, the majority were HuC/D^+^ and often displaced from the normal laminar position of existing HuC/D^+^ cells in the RGC and lower INL (Figures 3B′, S3B, and S3B′). Some of these were also colabeled with EdU confirming the genesis of new RGC-like cells (Figure S4B). Most lineage-traced cells in the ONL were HuC/D^+^ (Figure 3B′), although GFP^+^Otx2^+^ neurons were also observed (Figures S3C–S3C″). Overall, the cell fates of new neurons in the retinas of Ascl1-Atoh1 mice following light damage were ∼20% Otx2^+^ and ∼70% HuC/D^+^ (Figure 3C). The ratio of Otx2^+^/HuC/D^+^ neurons observed in the light-damaged retinas was similar, although not identical to that observed after NMDA damage, where ∼10% of new neurons were Otx2^+^ and the rest were HuC/D^+^ (Todd et al., 2021). Interestingly, most MG-derived neurons also expressed low levels of Sox2^+^ (Figures 3B′–3C), a phenomenon we have observed in previous studies, particularly at early time points in the reprogramming process (Todd et al., 2021; 2022).

To further investigate how injury mode may have influenced the fates of MG-derived neurons in Ascl1-Atoh1-expressing MG, we performed scRNA-seq on GFP^+^ sorted cells following the light-damage paradigm. The GFP^+^ cells from either NMDA-treated or light-damaged retinas were integrated and clustered into cell types based on their expression of known marker genes for these cell types (Figure 3E). We previously observed that reprogramming MG with these two TFs after NMDA injury leads to the majority of MG adopting an immature RGC fate. Our results following light-damage were very similar, with most MG-derived neurons expressing RGC markers (Figures 3E and 3F; pink) and only a few cells remaining MG (Figures 3E and 3F, yellow).

**Figure 4:**
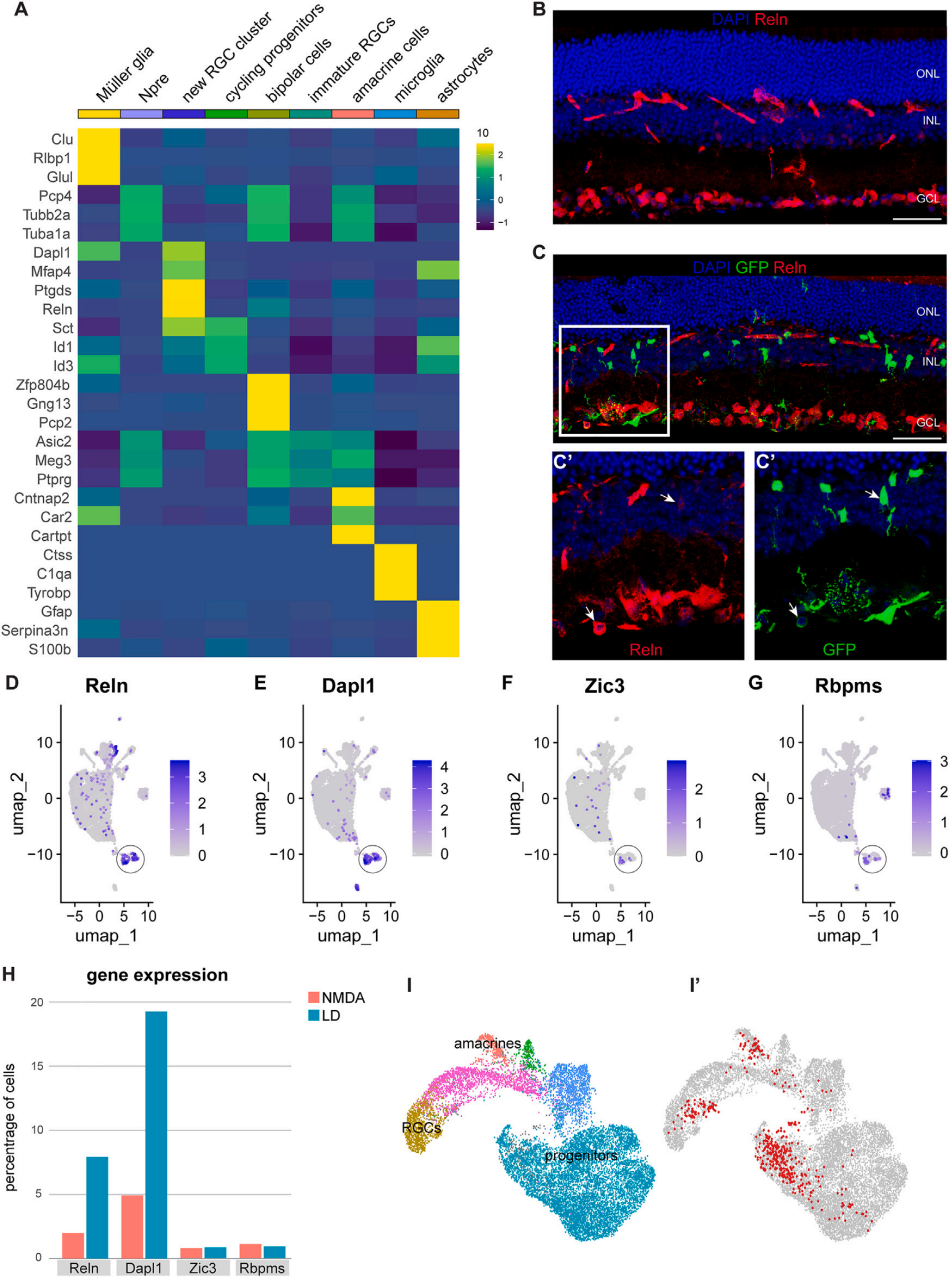
The impact of light damage on the transcriptome of reprogrammed MG with Ascl1-Atoh1 (A) Heatmap of the top 3 most differentially expressed transcripts for each cluster of the integrated Seurat object combining NMDA-treated and light-damaged GFP^+^ sorted cells. (B) Fluorescent image of retinal cross-section showing nuclear marker DAPI and Reln protein distribution in intact retina. (C–C′) Fluorescent image of retinal cross-section showing a light-damaged retina with lineage-traced GFP^+^Reln^+^ cells (white arrows). (D–G) Feature plot of (D) Reln, (E) Dapl1, (F) Zic3, and (G) Rbpms transcripts in the UMAP of integrated Seurat objects, with the new RGC cluster circled. (H) Quantification of cell percentage in each dataset expressing transcripts of genes for Reln, Dapl1, Zic3, and Rbpms, split by dataset origin. (I–I′) UMAP of integrated Seurat objects of E14 mouse retina and subset new RGC cluster showing (I) the main clusters of interest and (I′) where the new RGC cluster maps onto (red dots). Scale bar: 50 μm.

These results show that MG expressing Ascl1-Atoh1 will generate similar types of neurons after either inner or outer retinal damage. However, in addition to the bipolar and RGC-like neurons, we now observed the presence of a new cell cluster (black circle, Figures 3D–3D″). This cluster was mostly comprised of cells from the light-damaged dataset (black circle, Figure 3D″) and expressed the axon growth–associated gene Gap43, a marker of developing RGCs (Reh et al., 1993) (Figure 3G). To better define this new population, we plotted the top three differentially expressed transcripts across clusters (Figure 4A). This analysis revealed that the new RGC cluster was enriched for reelin (Reln), which is involved in circuit patterning and synaptic connectivity (Rice et al., 2001). In the uninjured adult retina, the Reln protein is expressed primarily by RGCs and at a lower level by amacrine cells in the INL (Figure 4B). After light-damage, we found some rare examples of lineage-traced GFP^+^Reln^+^ cells (Figures 4C–4C′), corroborating the scRNA-seq data that a new glia-derived RGC subtype was generated from MG expressing Ascl1-Atoh1 following light-damage (Figure 4D). The transcript for an important downstream component of the Reln-mediated signal transduction pathway, Disabled-1 (Dab1, Dapl1) (Rice et al., 2001), was also highly expressed in the new cluster (Figure 4E), as well as moderate expression of the RGC axon patterning gene Zic3 (Zhang et al., 2004) (Figure 4F) and the canonical RGC marker Rbpms (Figure 4G), confirming that these cells belong to the RGC lineage. Overall, there was a greater percentage of cells that expressed Reln and Dapl1 in the light-damaged dataset compared to NMDA, with similar levels for Zic3 and Rbpms across datasets (Figure 4H). To further investigate this new cluster with regards to transcript similarity with developing RGCs, we mapped the cluster onto a published scRNA-seq dataset of E14 mouse retina (Clark et al., 2019) when most RGCs are born (Figure 4I). We found that the cells clustered with developing progenitors, amacrine cells, and RGCs (Figure 4I′). It is important to note that the Reln^+^ RGC-like cells express transcripts for NMDA receptor genes (Figures S3D–S3G), and thus it is possible that the injury paradigm may influence the survival of these neurons. However, we consider this scenario unlikely, since NMDA receptor genes are expressed in all of the glia-derived RGCs (Figures S3D–S3G); therefore, such a phenomenon would have affected all of the RGCs, not just one class.

**Figure 5:**
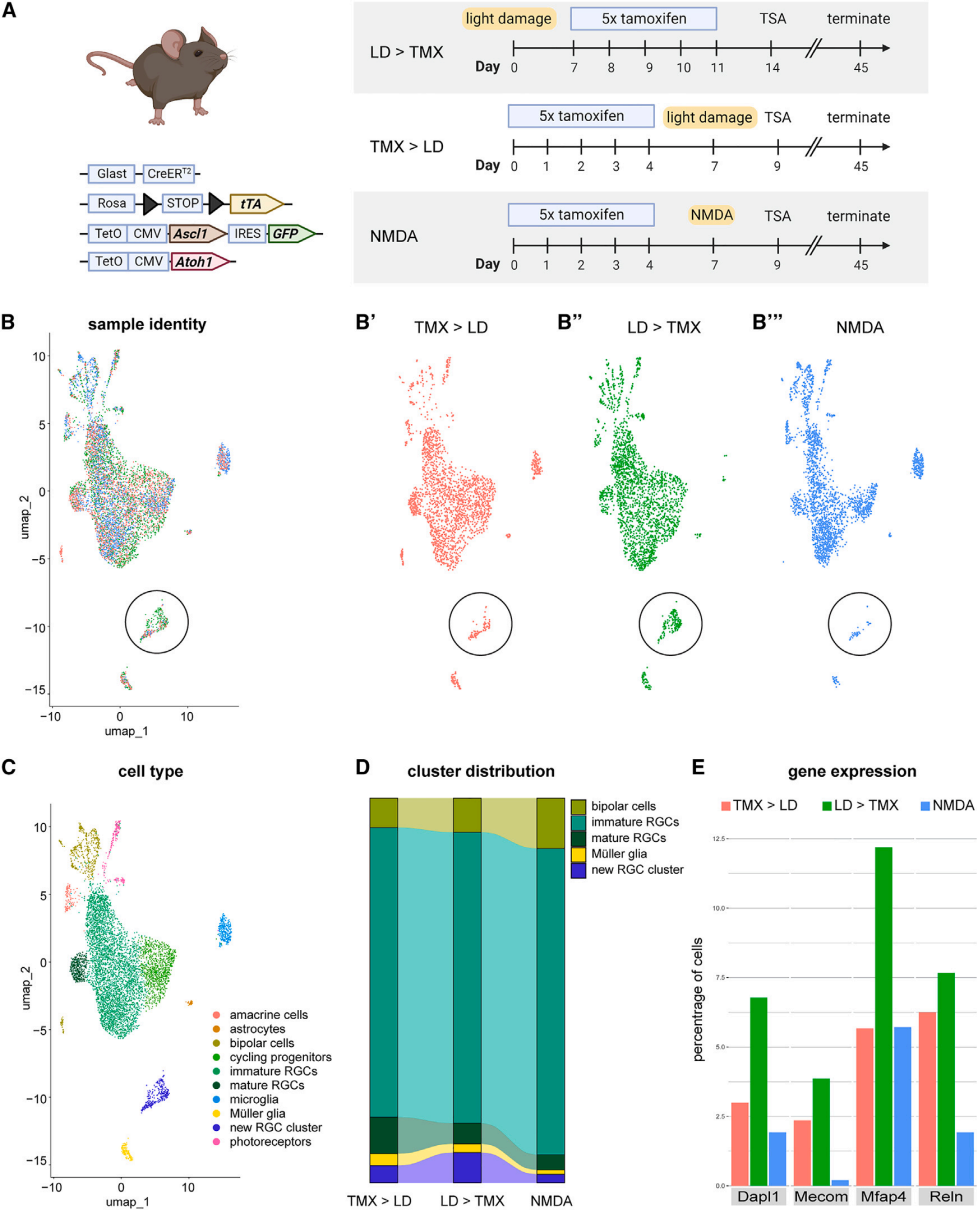
Investigating the effects of timing of light damage on reprogramming MG with Ascl1-Atoh1 (A) Schematic overview of the experimental time lines and injuries used to reprogram transgenic mice expressing Ascl1-Atoh1 specifically in MG. (B–B′) UMAP of integrated Seurat objects from sequencing runs of GFP+ sorted cells from light-damaged and NMDA-treated retinas showing the representation of each sample, (B′) light-damage after Ascl1-Atoh1 induction, (B″) light-damage before Ascl1-Atoh1 induction, and (B''') NMDA-damage. (C) UMAP of integrated Seurat objects split into clusters of cell types based on transcript signatures. (D) Alluvium plot of subseted cell types originating from the 3 datasets. (E) Quantification of cell percentage in each dataset expressing transcripts of Dapl1, Mecom, Mfap4, and Reln, split by dataset origin.

Obtaining a new neuronal subtype from MG expressing Ascl1-Atoh1 under different conditions of retinal damage suggests that the type of injury affects the cell fates generated by the MG-derived progenitors. However, the timing of the injury with respect to the TF expression was also varied in our experiments. To better compare the treatment conditions, we carried out an experiment with the same timeline that was used for the NMDA injury and instead performed light-damage—in other words, the TFs were expressed before the injury instead of after (Figure 5A). We harvested the GFP^+^ lineage-traced cells and ran scRNA-seq. The data from all of the conditions of retinal damage and timing (Figure 5A) were merged into a single UMAP (Figure 5B).

From this analysis, we observed that the new RGC population was primarily generated when light-damage preceded Ascl1-Atoh1 expression and to a lesser extent when light-damage followed TF expression (Figures 5B′–5B‴′, black circle). Interestingly, when light-damage was performed afterTF induction, we obtained a more mature RGC cluster from the MG-derived HuC/D^+^ RGCs, based on higher expression levels of markers such as Ebf2 (Figure 5C). Furthermore, the cluster distribution for MG and MG-derived neurons across the three datasets showed that performing light-damage after Ascl1-Atoh1 expression led to modest levels of Reln^+^ RGCs, but more mature Ebf2^+^ RGCs, whereas performing light-damage before Ascl1-Atoh1 expression produced the most Reln^+^ RGCs. NMDA damage yielded the fewest mature RGCs and Reln^+^ RGCs (Figure 5D), which was also reflected by the lowest gene expression levels of markers exclusive to the new RGC cluster (Figure 5E).

### Retinal inflammatory state can influence the formation of MG-derived neuron subtypes

Overall, our results demonstrate that MG expressing the combination Ascl1-Atoh1 can generate variable numbers of Reln^+^ RGCs depending on the injury mode and timing. This suggests that the retinal microenvironment after injury may affect the neurogenic process and ultimate fates of newborn neurons. One possible difference in the microenvironment of these different injury modes is the level of inflammation caused. At early time points following injury, the degree of inflammation and the signals received by MG may differ between injury modes, and this could influence the effects of TFs on stimulatedneurogenesis.

To investigate whether the mode of injury affects the level of inflammation in the retina, we assessed microglial localization in the retina at 2 and 4 days after light-damage (Figure S5A). We find that microglial presence in the damaged ONL is most prominent at day 4, when microglia infiltrate the thinning ONL as rods and cones gradually degenerate (Figures S5B and S5C). We also used CellChat (Jin et al., 2021) to ask whether there are differences in the signaling pathways that become engaged following light-damage and NMDA injuries, using published datasets of scRNA-seq mouse retina 72 h post-NMDA or light-damage (Hoang et al., 2020). Our analysis revealed that MG are the cells that receive the most signals from other cell types in the retina following injury (Figure S5D) and that NMDA evokes stronger inflammatory signaling in MG compared to light damage, including tumor necrosis factor (TNF), oncostatin-m (Osm), and angiopoietin-like 4 (Angptl4) (Figure S5E). The transcripts for these pathways were also expressed in our scRNA-seq data from Ascl1-Atoh1 reprogrammed retinas (Figure 5B), where NMDA injury led to higher levels of *Tnfa*, *Osm*, and *Angptl4* compared to light-damage, irrespective of timing (Figure S5F).

**Figure 6:**
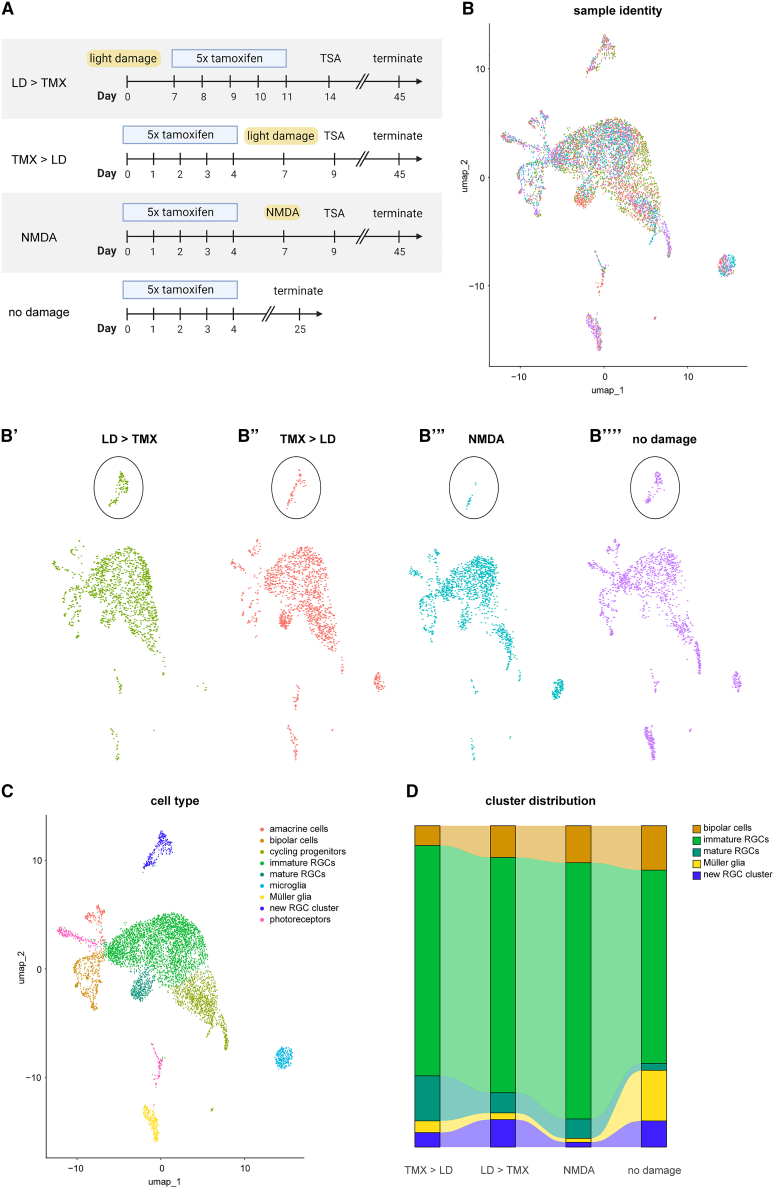
Investigating the effects of early inflammation on reprogramming MG with Ascl1-Atoh1 (A) Schematic overview of the experimental time lines and injuries used to reprogram transgenic mice expressing Ascl1-Atoh1 specifically in MG. (B–B‴′) UMAP of integrated Seurat objects from sequencing runs of sorted cells from all of the experimental retinas showing the representation of each dataset (B′) light-damage before Ascl1-Atoh1 induction, (B″) light-damage after Ascl1-Atoh1 induction, (B″′) NMDA damage, and (B‴′) no damage. (C) UMAP of integrated Seurat objects split into clusters of cell types based on transcript signatures. (D) Alluvium plot of subseted cell types originating from the 4 datasets split by dataset origin.

Taken together, it appears that the differences in neurogenesis from Ascl1-Atoh1-expressing MG as a function of injury mode and timing may be due to differences in the level of inflammation. If so, then reprogramming MG to a neurogenic state in the absence of retinal inflammation would also produce the newly identified Reln^+^ RGCs. When we first characterized the generation of neurons from MG expressing Ascl1-Atoh1, we noted that neurogenesis could be stimulated even without injury (Todd et al., 2021). This allowed us to test our hypothesis that MG inflammation affected the genesis of neuronal subtypes. We harvested GFP^+^ lineage-traced cells from uninjured animals 3 weeks after tamoxifen-induced Ascl1-Atoh1 expression and ran scRNA-seq. When we merged the Seurat objects from all four conditions (Figures 6A and 6B), it became evident that even in the absence of injury, there were cells clustering with the new RGC subtype (Figures 6B′–6B‴′, black circle). We obtained all of the clusters previously identified, including the glia-derived immature/mature RGCs, cycling progenitors, and bipolar cells (Figure 6C). Reln^+^ RGCs were generated from MG in the absence of injury at a level similar to that obtained when light-damage preceded Ascl1-Atoh1 expression, which was greater than both the NMDA and light-damage after TF induction (Figure 6D). A further prediction was that MG expressing Ascl1-Atoh1 in the undamaged retina would produce the fewest RGCs (HuC/D^+^) and the most unchanged MG compared to all of the injury conditions, and this was indeed the case (Figure 6D).

## Discussion

We set out to study whether the mode and timing of retinal injury affects the types of neurons generated by MG reprogrammed with proneural TFs in the adult mammalian retina. Our previous efforts to stimulate neurogenesis in the adult mouse retina demonstrated that Ascl1 induces neurogenesis from MG only when followed by inner retinal injury (Jorstad et al., 2017; Ueki et al., 2015). Studies in naturally regenerating species have suggested that the mode of injury or degree of cell death may affect the regeneration process (D’Orazi et al., 2016). Before the present study, the only mode of injury we had tested was a neurotoxic dose of NMDA delivered intravitreally, which kills RGCs and some amacrine cells. Moreover, we had primarily relied on a paradigm in which the neurogenic factors are induced in the MG before injury. Therefore, in this report we have explored whether timing or mode of injury affects the outcome of induced neurogenesis in MG using two different injury modes, two different timing protocols, and two different TF combinations.

Our results show that the mode of injury does not affect the outcome of reprogramming MG to Npre with Ascl1, since both light-damage and NMDA are sufficient to trigger neurogenesis. Based on histology, the ratio of glia-derived bipolar neurons in the light-damaged retinaswas comparable to that in the NMDA-treated retinas (Figure 1D), although scRNA-seq showed an increase in the rate of neurogenesis from the Ascl1-expressing MG after light-damage compared with that observed from NMDA injury (Figure 2D). The apparent discrepancy between the two outcome measures may be due to the sensitivity of each method, with histology reflecting the protein expression profiles and scRNA-seq providing a high-resolution overview of transcriptomic changes.

Although the overall rate of neurogenesis was very similar in both injury modes, we did observe a substantial difference in the laminar positioning of the MG and their neuronal progeny based on injury mode. Light-damaged retinas had more lineage-traced cells in the ONL (Figure 1D), including both MG-derived neurons (Otx2^+^) and potential MG-derived progenitors (Sox2+Otx2^−^) (Figures 1H–1H′). This phenomenon is reminiscent of the interkinetic nuclear migration of progenitors during retinal development (Baye and Link, 2008) and in the regenerating retinas of chicks (Fisher and Reh, 2000) and zebrafish (Lahne and Hyde, 2016), and it does not occur as frequently during reprogramming after NMDA damage (Figure 1D). This suggests that the loss of photoreceptors triggers a different response in the Ascl1-expressing MG, as opposed to RGC loss, which may be relevant to future studies in retinal repair.

We found that the mode of injury did not affect the types of neurons generated by the MG when the cells expressed Ascl1, since we obtained bipolar neurons regardless of the injury mode. Ablating photoreceptors in mice did not cause the Ascl1-expressing MG to generate new photoreceptors, indicating a lack of selective cell replacement controlled by the mode of injury. Although we detect some photoreceptors in our scRNA-seq data, we have not identified newborn photoreceptors via histology and EdU labeling, which is why we cannot exclude that these photoreceptors are carried over during fluorescence-activated cell sorting.

By contrast with the Ascl1 results, we found that the types of neurons generated from MG that express Ascl1-Atoh1 were sensitive to injury mode and timing. We identified a new type of RGC-like cell generated from MG expressing Ascl1-Atoh1 following light-damage, even though RGCs were not affected in this injury paradigm. Thus, although the type of injury can affect the cell types generated by the Ascl1-Atoh1-expressing MG, there does not appear to be selective cell replacement. This reinforces the notion that the environment of an injured retina has a limited effect on the cell fate of regenerated cells, and that in this system, fate is primarily driven by combinations of proneural factors.

The results we see in the mouse are not that different from what is observed in fish. Studies on regenerative species have used numerous injury paradigms to study retinal responses to global and layer- or cell-specific lesions reviewed by Sharma and Ramachandran (2022). In zebrafish, the early response to injury is similar regardless of injury mode, with reactive glia becoming proliferative and entering a neurogenic state (Hoang et al., 2020). Once reactive MG become multipotent progenitors in zebrafish, they give rise to all retinal neurons irrespective of injury, although they proliferate more in areas of cell death as opposed to areas where the retina is intact (Powell et al., 2016). In both zebrafish and mice, the transcriptomic profiles of resting and reactive MG are similar following injury (Hoang et al., 2020), suggesting that the greatest difference between regenerating and nonregenerating species is the capacity of MG to switch into proliferating progenitors, with less emphasis on the injury mode.

Some studies have reported that the ablation of specific neuron types can bias the fate of regenerated neurons toward the cell type lost. For example, selective ablation of bipolar neurons in zebrafish led to the regeneration of mostly bipolar neurons, with some new rods and cones also born following injury (D’Orazi et al., 2016). A similar bias was reported when cones were ablated in zebrafish, with the selective loss of red and UV cones also eliciting the regeneration of those same subtypes (D’Orazi et al., 2020). It is important to note, however, that not all injury paradigms are equal even in regenerative species, because the selective ablation of blue cones in zebrafish could not stimulate their regeneration (D’Orazi et al., 2020).

In addition to the effects on cell fate, different types of injury could control other aspects of the regeneration process. It has been suggested that the degree of cell death can be sensed by the retina and as a result, lead to varying pathways of regeneration and cell replacement (Montgomery et al., 2010). As noted above, MG-derived progenitors in zebrafish proliferate more in areas of cell death than in areas where the retina is intact (Powell et al., 2016). In the present study, we did not detect regional differences in the proliferation of the reprogrammed MG, due to the broad effects of both light-damage and NMDA across the retina.

Our data have shown that photoreceptor ablation triggers MG expressing Ascl1-Atoh1 to generate RGC-like cells and specifically a new subclass of Reln^+^ RGC-like neurons. Reln is a secretory protein in the CNS that facilitates the migration of neurons to their final laminar position in the brain during development (Rice and Curran, 2001). It has a similar role in the retina, in which at embryonic day 13.5, it is predominantly expressed by neurogenic precursors, RGCs, and the distal ciliary margin (Broad Institute Single Cell Portal analysis of data from Balasubramanian et al., 2021) serving as a guidance cue for synaptic connectivity within the retina (Rice et al., 2001).

Although Reln and its downstream effector protein Dapl1 (or Dab1) are primarily expressed by RGCs and glycinergic AII amacrine cells, respectively (Rice and Curran, 2000; Rice et al., 2001), Reln signaling has also been implicated in the rod photoreceptor pathway. Mice deficient in either Reln or Dab1 have fewer rod bipolar cells and attenuated scotopic responses (Rice et al., 2001), indicating that Reln signaling is required for both the circuit formation and function of rod photoreceptors. It is possible that the ablation of rods after light-damage triggers the expression of signals naturally required for rewiring the remaining rods with the surviving neural network.

In addition to external cues that may bias the fate of regenerating neurons, we find that the inflammatory state of the MG has an effect on the types of neurons generated. MG expressing Ascl1-Atoh1 were able to generate Reln^+^ RGCs in the absence of any injury and when light-damage preceded TF expression by 1 week. When retinas were injured either with NMDA or light-damage after TF expression, the population of Reln^+^ RGCs was reduced (Figure 6). This suggests that the types of neurons generated by the reprogrammed MG depends on the degree of inflammatory signals they receive, since the proinflammatory signals they receive early following retinal injury are greater with NMDA than with light-damage (Figure S5).

We also assessed how the timing of injury would affect the regenerative capacity of mammalian MG in our models. We saw that photoreceptor ablation with light-damage before or after Ascl1 expression in MG was sufficient stimulus to elicit comparable levels of neurogenesis (Figure S2). The same result was obtained from MG expressing ASscl1-Atoh1 either before or after light-damage, although this was not surprising considering this TF combination can induce neurogenesis even without injury (Figure 6; Todd et al., 2021). This has important implications for the translation of this approach into a regenerative therapy for retinal disease, because patients would require cell replacement after photoreceptor degeneration.

We are now just beginning to understand the multifaceted process of induced regeneration in the mammalian retina. This work serves as basis for future investigations on how the environment can influence the efficiency of a regenerative regimen and inform subsequent attempts to treat diseases where specific neuron types are lost.

## Experimental procedures

### Resource availability

#### Corresponding author

Further information and requests for resources and reagents should be directed to and will be fulfilled by corresponding author Thomas A. Reh: tomreh@uw.edu.

#### Materials availability

No new reagents were generated for this work.

#### Data and code availability

All of the scRNA-seq datasets generated for this manuscript have been deposited in the Gene Expression Omnibus (GEO) repository under the accession number GSE250019.

### Animals

All of the animals were treated and housed with University of Washington Institutional Animal Care and Use Committee–approved protocols. Transgenic mouse lines Glast-CreER^t2^:LNL-tTA:tetO-Ascl1-IRES-GFP and Glast-CreER^t2^:LNL-tTA:tetO-Atoh1:tetO-Ascl1-IRES-GFP were previously characterized (Jorstad et al., 2017; Todd et al., 2021). Males and females were both used in experiments at equal frequencies. All of the experiments were performed on adult mice that were older than 30 days. For EdU incorporation, animals were given 0.4 mg/mL EdU/H_2_O *ad libitum* from the first day of intraperitoneal tamoxifen injections until the animals were sacrificed.

### Light damage

Mice homozygous for leucine at residue 450 of RPE65 were placed in cages covered in aluminized polyethylene mylar under white light-emitting diode bulbs. Light intensity was measured using a lux meter (LX1330B digital illuminance light meter) to verify that ∼10,000 lux was emitted within the cages. Animals received eye drops of tropicamide ophthalmic solution to dilate their pupils right before illumination. Each cage housed 1–2 animals for a period of 6 h. Mice were monitored for photoaversion to ensure sufficient light exposure.

### Injections

Intravitreal injections were performed with a 32G Hamilton syringe (1 μL volume) on mice anesthetized with isoflurane. Concentrations were 100 mM for NMDA/PBS and 1 μg/μL TSA/DMSO. Intraperitoneal injections of tamoxifen (1.5 mg/100 μL of corn oil) were administered daily for 4–5 consecutive days.

### Immunohistochemistry

Dissected eye cups were fixed with 4% paraformaldehyde/PBS for 30 min and then incubated in 30% sucrose solution at 4°C overnight. Eyes were then embedded in optimal temperature cutting compound before freezing. Frozen samples were sectioned at −20°C in 15- to 18-μm sections onto glass slides. Slides were then heated for 10 min on a slide warmer before staining or freezing at −20°C for long-term storage.

For staining, slides were traced with a liquid blocker pen and then rehydrated with PBS. Primary antibodies were incubated overnight at 4°C in blocking solution (0.3% Triton X-100 and 5% normal horse serum in PBS). The primary solution was removed, and slides were washed with PBS. Secondary antibodies were incubated in blocking solution for 90 min and then slides were washed with PBS. Fluoromount-G (SouthernBiotech) mounting medium was added to slides before covering with a glass coverslip. See Tables S1 and S2 for all of the antibodies used.

### Microscopy

Sections were imaged with a Zeiss LSM880 microscope. Images were taken with a 20× objective, with at least 3 images taken per retina for quantification. Images were then analyzed and counted using FIJI (Schindelin et al., 2012).

### FACS

Following euthanasia, pools of four retinas were dissociated using the Worthington Papain Dissociation System (catalog no. LK003150) according to the manufacturer’s instructions. Cells were then spun at 4 °C at 400 × *g* for 10 min and resuspended in neurobasal medium (Gibco no. 21103049), 10% fetal bovine serum (Clontech), B27 (Invitrogen), N2 (Invitrogen), 1 mM l-glutamine (Invitrogen), and 1% penicillin-streptomycin (Invitrogen). The cell suspension was passed through a 35-μm filter and then sorted using a BD FACSAria III cell sorter (BD Bioscience) to retrieve all GFP^+^ cells. During sorting, appropriate gates were implemented to exclude debris, doublets, and autofluorescent cells. Using the purity settings, a minimum of 40,000 events were collected in 1.7 mL Eppendorf tubes that were previously coated with 10% BSA.

### scRNA library construction

FACS-purified GFP^+^ MG cells were spun at 4 °C at 400 × *g* for 10 min and resuspended at a concentration of 1,000 cells/μL. Library construction was performed using the Chromium Next GEM Single Cell 3′ version 3.1 (dual index) protocol and reagents according to the manufacturer’s instructions.

### scRNA-seq, mapping, and data analysis

Multiplexed libraries were sequenced using an Illumina NextSeq 500 using high-output 150 kits. Data were demultiplexed and aligned to the mouse mm10 genome using CellRanger version 3.0.2 (Zheng et al., 2017). Filtered output files were further analyzed in R using Seurat version 4.3.0 (Hao et al., 2021), ggplot2, data.table, dplyr, tidyr, and other commonly used R packages. Low-quality cells (identified as having low read depth or high mitochondrial content; >10%) were removed from datasets. Before analysis, the cell number was downsampled to the object with the lowest cell number and cell unique molecular identifiers were downsampled to the lowest median for each object using the package scuttle’s downsampleMatrix function. Cells were clustered using principal-components analysis and UMAP. Comparisons between datasets were made by canonical correlation analysis, as described by the Satija laboratory vignette (https://satijalab.org/seurat/archive/v3.0/integration.html). Alluvial bar plots were created by the percentage of cells in each cluster split by each sample using ggplot2 with ggalluvial. Heatmap was calculated using Seurat’s DoHeatmap over the top three differentially expressed (DE) genes for each cell type cluster with their average expression of that gene. The top 3 DE genes were found by using FindAllMarkers and sorted by the average log_2-fold change for each single cluster compared against all others. See Table S3 for Cell Ranger summaries.

### Integration with development data

For comparison to developing retina, data were first downloaded from the GEO (Clark et al., 2019). The label transfer was carried out in Seurat using a reference dataset composed of 432 randomly sampled cells of each major cell class in the developing retina dataset, for a total of 3,024 cells, as identified by canonical markers. Reads were downsampled to a common average depth before analysis. The Reln^+^ cluster was subset and integrated directly with a subset composed only of embryonic day 14 (E14) cells from the development dataset.

### CellChatDB analysis

To identify differences in the incoming signals that MG receive immediately after injury, either NMDA or light damage, published scRNA data from mouse retina were first downloaded from GEO (Hoang et al., 2020). We then used CellChatDB (Jin et al., 2021) to compare the expression of signaling ligands, receptors, soluble agonists, antagonists, and co-receptors between the datasets from light-damaged or NMDA-injured retinas and retinal cell populations to identify differentially expressed signaling pathways. Using the inbuilt network analysis and pattern recognition tools, we identified candidate signaling pathways that MG receive input from following injury.

## Supplemental information

Document S1. Figures S1–S5 and Tables S1–S3

Document S2. Article plus supplemental information
