# Supplementary material for: The impact of timing and injury mode on induced neurogenesis in the adult mammalian retina
Source: Stem Cell Reports. 2024 Jan 25;19(2):239–53. doi: 10.1016/j.stemcr.2023.12.010 (PMC10874861; doi:10.1016/j.stemcr.2023.12.010)
Supplement: Document S1. Figures S1–S5 and Tables S1–S3 [file mmc1.pdf]

Stem Cell Reports, Volume *19*

Supplemental Information

The impact of timing and injury mode on induced neurogenesis in the adult mammalian retina

Marina Pavlou, Marlene Probst, Nicolai Blasdel, Aric R. Prieve, and Thomas A. Reh

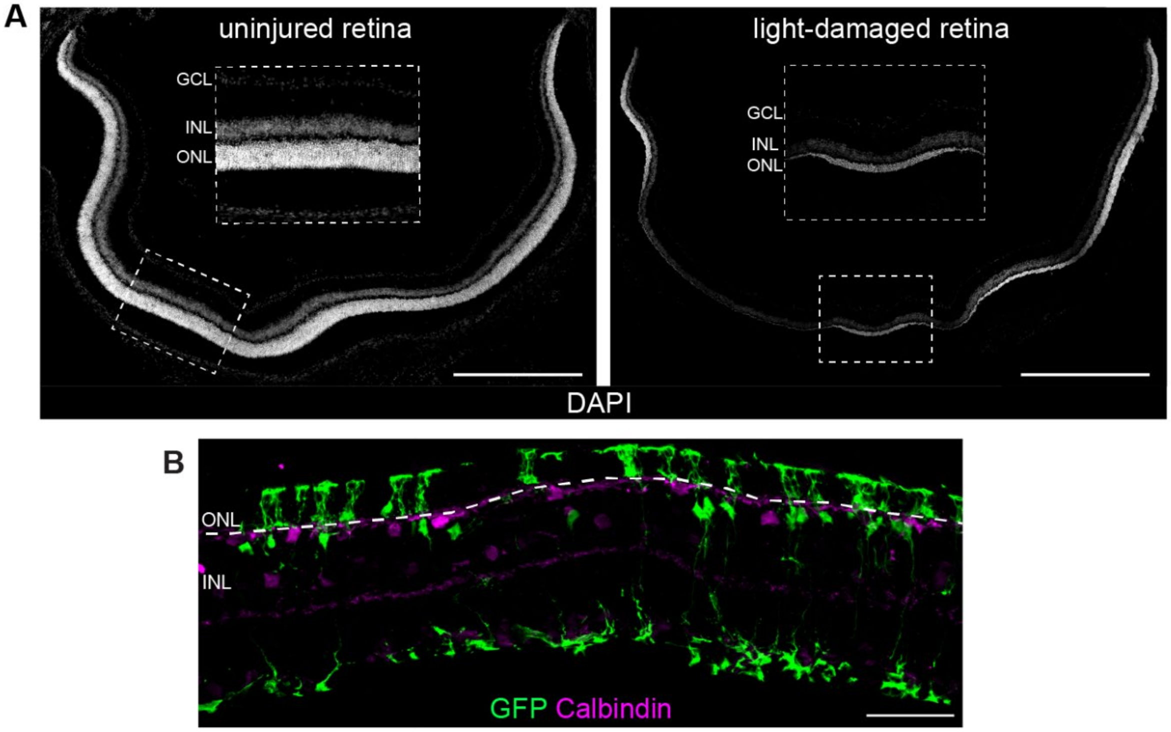

Supplementary Figure 1: the impact of light-damage on reprogramming MG with Ascl1; (A) fluorescent images of retinal cross-sections stained with nuclear marker DAPI from an injured retina and a light-damaged retina 7 days post injury; (B) fluorescent image of retinal cross-section showing lineage-traced GFP+ on ONL and INL margin (white line) not labelled with Calbindin. Scalebar A: 500µm, B: 50µm, ONL= outer nuclear layer, INL = inner nuclear layer, GCL= ganglion cell layer

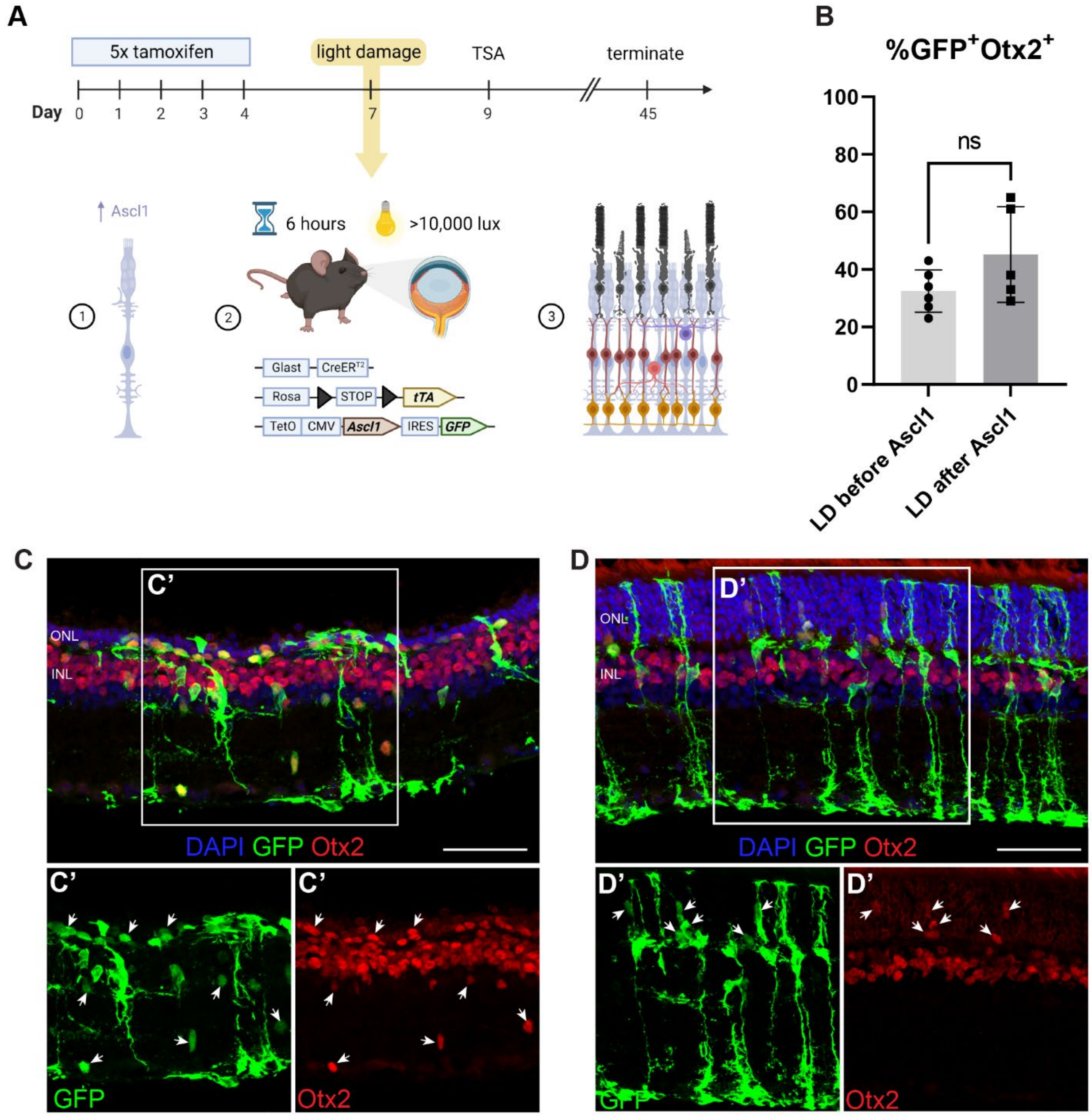

Supplementary Figure 2: the impact of injury timing with respect to MG reprogramming with Ascl1; (A) schematic overview of the experimental timeline where transgenic mice expressing Ascl1 specifically in MG in a tamoxifen-inducible manner undergo light-damage; (B) quantification of double-labelled GFP+Otx2+ cells after light-damage before or after inducing Ascl1 expression in MG; (C-C') fluorescent images of retinal cross-section showing examples of lineage-traced GFP+Otx2+ cells in area of severe ONL thinning (white arrows) and (D-D') less severe ONL thinning (white arrows) after light-damage. Scalebar: 50µm, bar graphs (n≥5 animals) with SEM error bars and unpaired t-test analysis, ns=not significant.

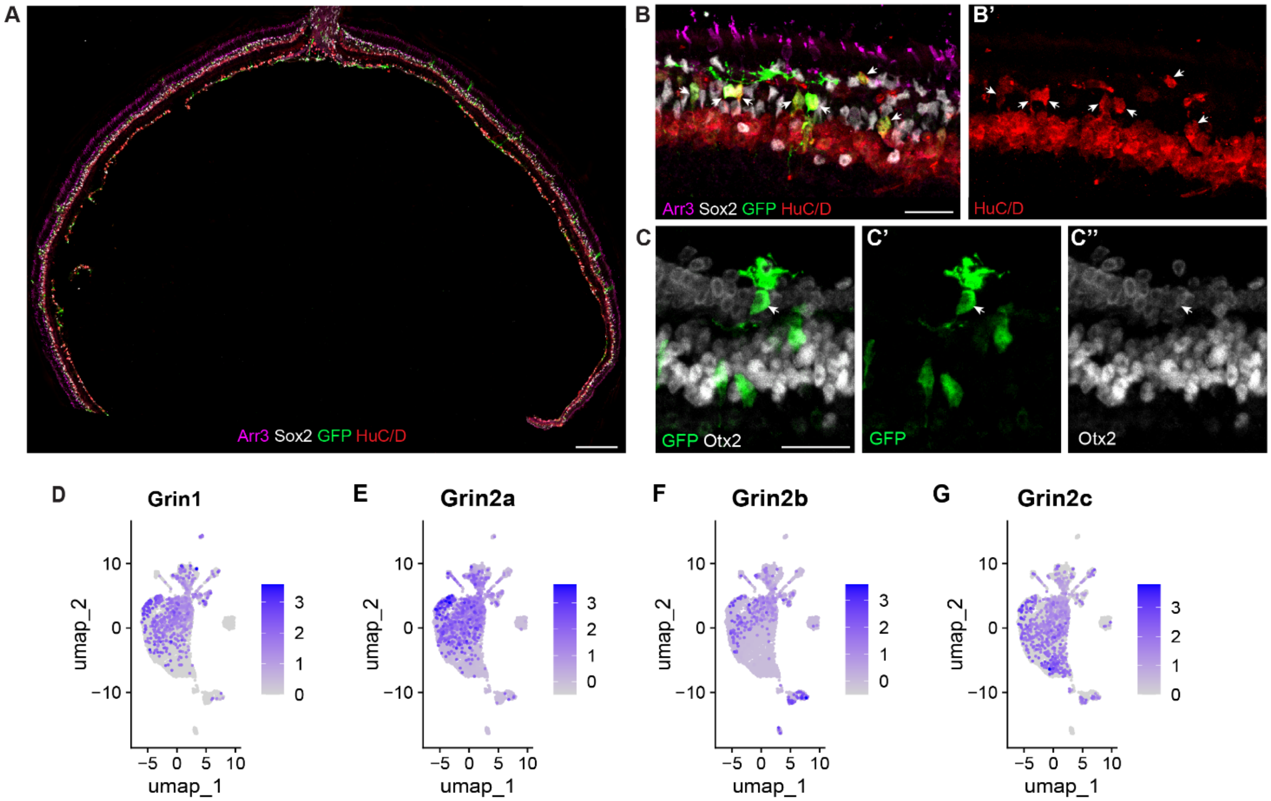

Supplementary Figure 3: the impact of light-damage on reprogramming MG with Ascl1-Atoh1; (A) fluorescent image of retinal cross-section showing an overview of the central retina after light-damage; (B-B') fluorescent images of retinal cross-section showing lineage-traced cells with GFP co-labelled with HuC/D (white arrows); (C-C'') fluorescent images of retinal cross-section showing lineage-traced cell with GFP co-labelled with Otx2 (white arrow). (D-G) feature plots of NMDA receptor transcripts in the UMAP of integrated Seurat objects from sequencing runs of sorted cells from light-damaged and NMDA-treated retinas. Scalebar A: 500μm, B-C: 25μm

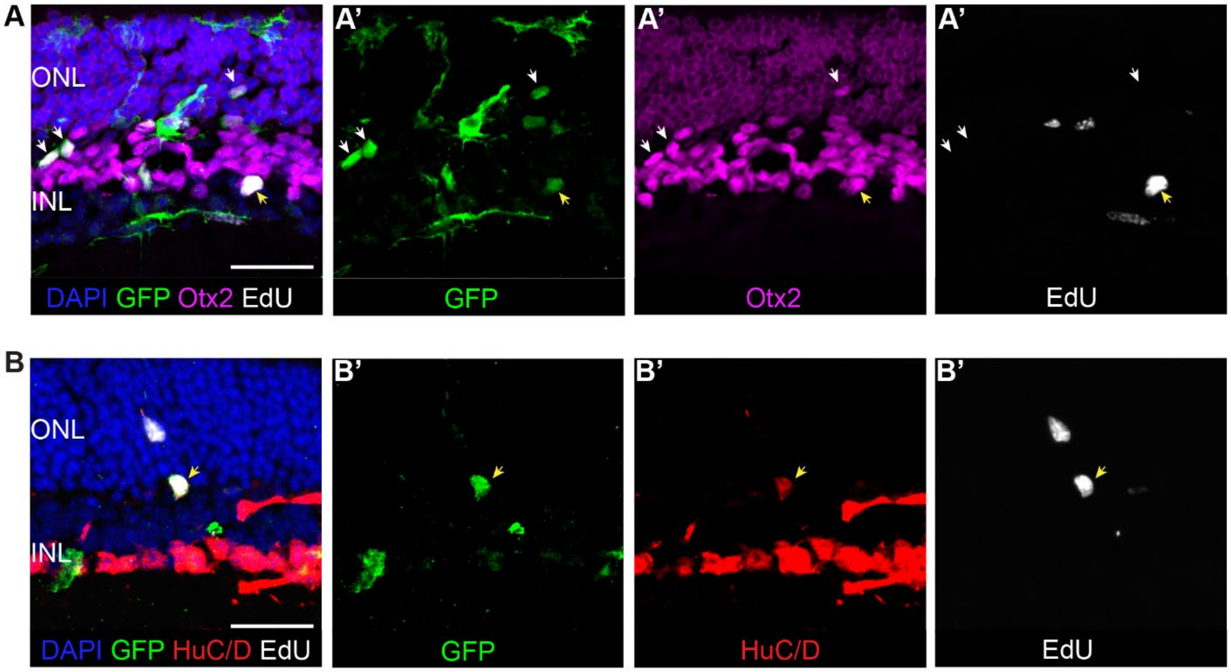

Supplementary Figure 4: reprogramming after light-damage leads to bona fide new neurons; (A-A') fluorescent images of retinal cross-section showing lineage-traced cells with GFP co-labelled with Otx2 (white arrows) and also EdU (yellow arrow); (B-B') fluorescent images of retinal cross-section showing lineage-traced cells with GFP co-labelled with HuC/D and EdU (yellow arrows). Scalebar: 25μm

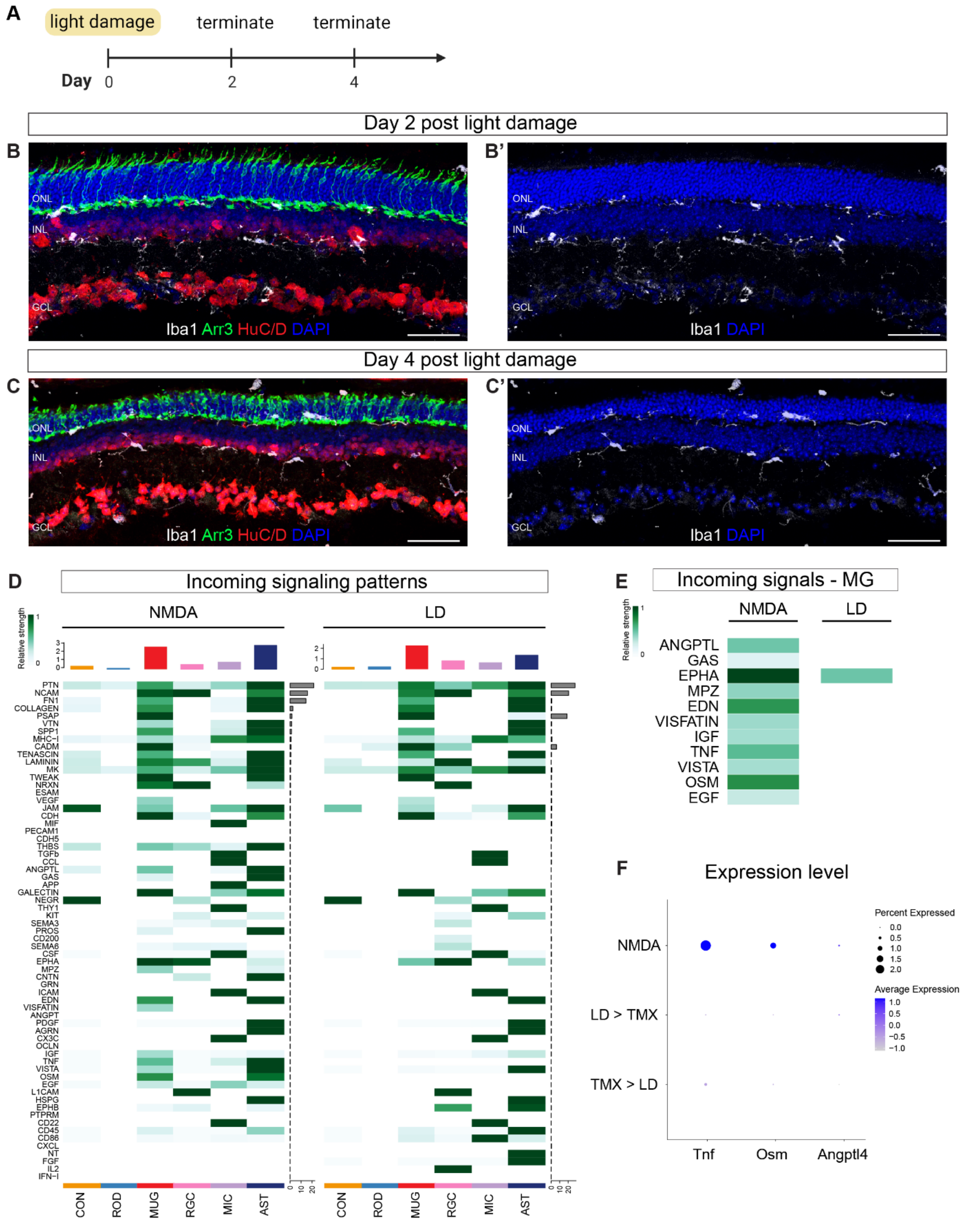

Supplementary Figure 5: inflammation at early timepoints after retinal injury; (A) schematic overview of the experimental timeline; (B-C) fluorescent images of representative cross-sections showing ONL integrity and microglia (Iba1) infiltration 2 days (B) and 4 days (C) after light-

damage; (D) CellChat analysis of published scRNA-seq dataset from Hoang *et al* 2020 showing heatmap of incoming signaling patterns (y-axis) received by main retinal cell types (x-axis) following either NMDA-damage or light-damage; (E) focused heatmap showing only differentially activated signaling patterns received by Müller glia after NMDA-damage or light-damage; (F) dotplot showing the expression level of pro-inflammatory transcripts across Ascl1-Atoh1 reprogramming datasets following the different injury modes.

Supplementary table 1: Primary Antibodies

**Antibody**	**Source**	**Identifier (Cat#)**	**Concentration**
rabbit anti-Arrestin3	Abcam	AB15282	1:200
rabbit anti-Calbindin	Millipore	AB1778	1:1000
rabbit anti-GFAP	Dako	Z0334	1:500
chicken anti-GFP	Abcam	AB13970	1:1000
mouse anti-HuC/D	Invitrogen	A-21271	1:200
goat anti-Otx2	R&D Systems	BAF1979	1:500
mouse anti-Pcp2	Santa Cruz	SC-137064	1:500
rabbit anti-Recoverin	Millipore	AB5535	1:500
mouse anti-Reelin	Abcam	AB78540	1:500
rabbit anti-Secretagogin	Cell Signaling Technology	14037	1:300
goat anti-Sox2	Santa Cruz	SC-17320	1:200

Supplementary table 2: Secondary Antibodies

**Antibody**	**Source**	**Identifier (Cat#)**	**Concentration**
donkey anti-chicken 488	Jackson Immuno	703-545-155	1:500
donkey anti-goat 405	Invitrogen	A48259	1:500
donkey anti-goat 568	Life Technologies	A11057	1:500
donkey anti-goat 647	Jackson Immuno	705-605-147	1:500
donkey anti-mouse 568	Life Technologies	A10037	1:500
donkey anti-mouse 647	Jackson Immuno	715-605-150	1:500
donkey anti-rabbit 568	Life Technologies	A100042	1:500
donkey anti-rabbit 647	Thermo Fisher Scientific	A-31573	1:500

Supplementary table 3: scRNA-seq cell ranger output summaries

	**Light-damage Ascl1**	**NMDA Ascl1**	**Light-damage before Ascl1-Atoh1**	**NMDA Ascl1-Atoh1**	**Light-damage after Ascl1-Atoh1**	**No damage Ascl1-Atoh1**
Estimated cell number	5,699	3,248	6,614	3,636	3,573	1,614
Mean reads per cell	2,766	47,639	6,850	27,070	10,320	31,894
Valid Barcodes	96.4%	95.4%	96.8%	97.2%	95.3%	96.5%
Median UMI counts per cell	1,023	4,770	1,941	3,968	2,998	3,100
Median genes per cell	709	2,042	1,244	1,981	1,791	1,668
Total genes detected	21,142	20,257	22,705	19,599	22,298	19,467
